# Lignin Biopolymer for the Synthesis of Iron Nanoparticles and the Composite Applied for the Removal of Methylene Blue

**DOI:** 10.3390/polym13213847

**Published:** 2021-11-07

**Authors:** Fang-Yi Peng, Pei-Wen Wang, Weisheng Liao, Ing-Song Yu

**Affiliations:** Department of Materials Science and Engineering, National Dong Hwa University, Hualien 97401, Taiwan; 410522001@gms.ndhu.edu.tw (F.-Y.P.); 410722059@gms.ndhu.edu.tw (P.-W.W.); liao1427@alumni.uidaho.edu (W.L.)

**Keywords:** lignin, zero valent iron, nanoparticles, ethylene glycol, methylene blue

## Abstract

In the current study, lignin, an abundant natural polymer, was dissolved in ethylene glycol and acidic H_2_O to form nanoscale lignin. Then, zero-valent iron (ZVI) nanoparticles were synthesized in nanoscale lignin, producing a nZVI/n-lignin composite, via the borohydride reduction method. The use of nZVI/n-lignin for environmental remediation was tested by the removal of methylene blue in aqueous solutions at room temperature. The nZVI/n-lignin composite achieved a higher methylene blue removal ratio than that achieved by traditional nZVIs. Moreover, its excellent dispersibility in water and stability against oxidation in the air were observed. The functions of the nanoscale lignin in the composite material are (1) prevention of further growth and aggregation of the nZVI nanoparticles, (2) protection of nZVI from serious oxidation by H_2_O/O_2_, and (3) allowing better dispersibility of nZVI in aqueous solutions. These three functions are important for the field applications of nZVI/n-lignin, namely, to travel long distances before making contact with environmental pollutants. The present method for producing nZVI/n-lignin is straightforward, and the combination of nZVI and lignin is an efficient and environmentally friendly material for environmental applications.

## 1. Introduction

The utilization of nanoscale zero-valent iron (nZVI) for in situ remediation of contaminated water and soil has been an active research area due to its low cost, high reactivity, good mobility, and environmental compatibility [1,2,3]. The synthesis of nZVI can be performed by top-down or bottom-up methods, such as the ball milling or lithography grinding of bulk iron materials, the hydrogen reduction of goethite/hematite at elevated temperatures, carbothermal reduction, electrolysis, pulsed plasma in liquids, and the reduction of ferric/ferrous salts in H_2_O by polyphenolic plant extracts or other reducing agents [4]. Among these, the sodium borohydride reduction of iron salts in H_2_O is the most widely studied method for the synthesis of reactive nZVI in academic research due to its simplicity [5]. The particle size of nZVI synthesized via the borohydride method usually ranges from ten to hundreds of nanometers, and it tends to gather into linear or fractal patterns because of its magnetic interaction, high surface energy, and van der Waals forces, which decreases its reactivity and mobility [6]. In addition, nZVI possesses a core–shell structure in which the surface is a thin layer of defective iron oxide that forms spontaneously during synthesis and continuously develops [5]. Therefore, using an inert environment during synthesis to prevent substantial oxide layer formation and modification of the nZVI surface to prevent particle aggregation is of great importance for the environmental applications of nZVI.

Ethylene glycol (EG) is an important raw material mainly used for the manufacture of polyester fibers and antifreeze, which can be mixed with H_2_O at any ratio. Synthesis of nZVI using the borohydride method in H_2_O with a small amount of EG can eliminate the need for an inert nitrogen or argon atmosphere and make the synthetic process simpler and less costly [7]. EG-functionalized nZVI provides better stability against oxidation and higher dispersibility in H_2_O than those of bare nZVI. The particle sizes of EG-functionalized nZVI are normally in the range of 20 to 100 nm, which are slightly smaller than those of bare nZVI synthesized by the borohydride method. Moreover, a novel flowerlike Fe-EG nanostructure has also been synthesized by dissolving FeCl_3_·6H_2_O and CH_3_COONa·3H_2_O in EG, following a thermal treatment method [8]. This polymeric ferrous glycolate can play the role of a Fenton catalyst in the wastewater treatment aspect of environmental remediation.

To prevent the aggregation of nZVI, various modification methods have been proposed, such as the deposition of other metals on its surface (e.g., Pd or Ni), coating nZVI with stabilizers (e.g., starch, guar gum, carboxymethyl cellulose, or polyacrylic acid), emulsifying nZVI, and producing composite materials of nZVI in a matrix (e.g., silica, CaCO_3_, montmorillonite, granular activated carbon, or carbon nanotube) [9]. In addition, supporting the nanoparticles with sustainable biopolymers could increase their stability and biocompatibility, as well as allowing them to attach different ligands to their surfaces [10]. Lignin-modified nZVI with a high specific surface area was first synthesized via the borohydride method in H_2_O for the removal of arsenic from groundwater [11]. Recently, bentonite-supported organosolv lignin-stabilized nZVI was fabricated for the removal of hexavalent chromium from wastewater [12].

Lignin is a heterogenous phenylpropanoid macromolecule with a three-dimensionally branched structure that includes random crosslinks of monomeric units [13]. It is also the second most abundant biopolymer, formed by the free radical polymerization of three monolignols, coniferyl alcohol (G), sinapyl alcohol (S), and *p*-coumaryl alcohol (H) in the plant cell wall, giving it a structure containing many aliphatic and aromatic hydroxyls [14]. The β-O-4 ether linkage generally accounts for up to 50% of all the linkages in a lignin network. The paper industry generates large amounts of lignin (or Kraft lignin) as a major by-product, and it is mainly used as fuel [15]. Due to its non-toxicity, biodegradability, renewability, and its 3D network containing hydroxyl groups, lignin has also been used as a stabilizing and/or reducing agent in the synthesis of Pt [16], Pd [17,18], Ru, Re [19], Au [20], and Ag [21,22] nanoparticles for cross-coupling reactions, oxidation/reduction reactions, detection of Pb^2+^, and as antibacterial materials.

There is global awareness of limited resources, and so the development of sustainable materials for reuse and recycling is urgent [23]. In this study, Kraft lignin was used as a starting material alongside various other types of lignin due to its easy availability and high abundance as a by-product of the paper industry. The particle size of Kraft lignin is usually in the micro-sized range [24], which still presents challenges for polymer blending or use as a solid support for metal nanoparticles. Since Kraft lignin is only soluble in H_2_O at pH > 10, while it is soluble in EG at up to 70 wt% [25], EG was selected to dissolve the Kraft lignin, which could subsequently be converted to lignin nanoparticles simply by using the antisolvent or acid precipitation method [26,27]. Therefore, nanoscale lignin was synthesized by dissolving commercial micro-sized Kraft lignin in EG, which was then added to acidic H_2_O containing iron salts via the antisolvent and acid precipitation methods. Afterward, the iron ions trapped in the nanoscale lignin were reduced to nZVI by the borohydride method. By using the nanoscale lignin to stabilize the nZVI, the resulting composite, called nZVI/n-lignin, may possess a higher colloidal stability and better mobility than that of the bare nZVI and the EG-functionalized nZVI. Characterizations of nZVI/n-lignin were performed by transmission electron microscopy, X-ray photoelectron spectroscopy, and an X-ray diffractometer. The dispersibility in water and stability against oxidation of nZVI/n-lignin were compared with those of bare and EG-functionalized nZVIs. Finally, the effectiveness of nZVI/n-lignin for the removal of methylene blue in H_2_O was investigated, and the process was monitored via the characteristic UV–Vis absorption peaks of the cationic dye. This nZVI/n-lignin composite material could be employed for application in environmental remediation in the future.

## 2. Materials and Methods

### 2.1. Chemicals

Iron (III) chloride hexahydrate (FeCl_3_·6H_2_O, ≧99%), Kraft lignin, ethylene glycol (EG), sodium borohydride (NaBH_4_, ≧98%), and ethanol were purchased from Sigma-Aldrich (Milwaukee, WI, USA). Methylene blue solution (0.1%) was supplied by Choneye Pure Chemicals (Tamil Nadu, India).

### 2.2. Preparation of Nanoscale Lignin-Stabilized nZVI

**Figure 1:**
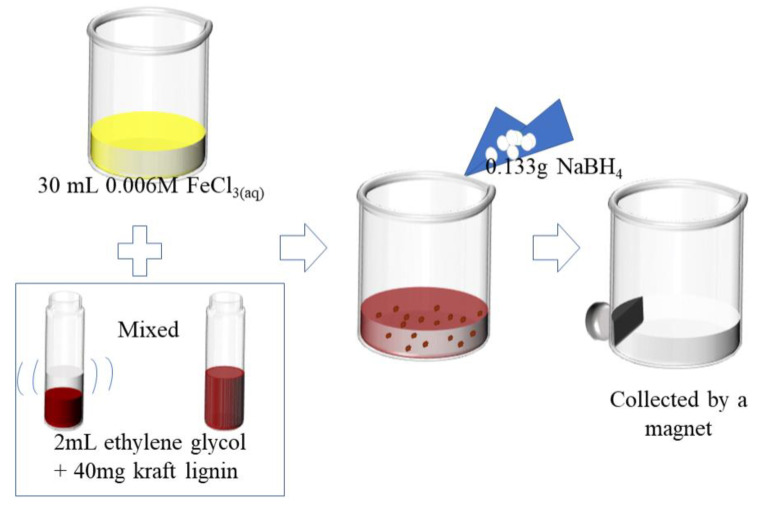
Experimental procedure for the synthesis of nZVI/n-lignin composite material.

The experimental procedure is shown in Figure 1. An amount of 40 mg of Kraft lignin was first dissolved in 2 mL EG to form nano-sized lignin, which could be observed by transmission electron microscopy. The dissolution process happened quickly, producing a brownish EG solution. After the dissolution, the EG solution containing the dissolved Kraft lignin was added to a 30 mL aqueous solution alongside 0.006 M FeCl_3_. After the solution had been mixed for 10 min, 0.133 g NaBH_4_ was added to it. Tiny black particles displaying magnetic behavior were formed quickly. After a short period, the DI-H_2_O-containing EG and nZVI/n-lignin were separated by a magnet. The nZVI/n-lignin composite was washed with ethanol three times. The washed nZVI/n-lignin was dried in an oven at a temperature of 40 °C for 20 min. Using the same method, the bare nZVI and EG-functionalized nZVI were also synthesized for comparison with nZVI/n-lignin composite. These nZVIs were then stored under atmospheric conditions for the subsequent characterizations and the degradation experiment in methylene blue solution. Bare nZVI, EG-functionalized nZVI, and nZVI/n-lignin were characterized by transmission electron microscopy (TEM, JEOL JEM-2010, Tokyo, Japan), X-ray diffractometer (XRD, Rigaku D/MAX-3C OD-2988N, Neu-Isenburg, Germany), and X-ray photoelectron spectroscopy (XPS, Thermo Scientific K-Alpha, Waltham, MA, USA). The XPS depth profile analyses were also conducted by argon ion etching using an energy level of 3000 eV for 1 min.

### 2.3. Dispersibility and Stability against Oxidation of nZVI/n-Lignin

To verify the applicability of nZVI/n-lignin, the synthesized bare nZVI, EG-functionalized nZVI and nZVI/n-lignin were tested in the aqueous solutions for 7 days. The dispersibility of the two nZVIs and nZVI/n-lignin in H_2_O was observed. Moreover, their stability against oxidation in air and water was verified. After the synthesis of materials, the three dried materials were stored in atmospheric conditions for 7 days and placed in water for 7 days. Then, the powders were measured by XRD to check the characteristic peaks of zero-valent iron and iron oxides.

### 2.4. Removal of Methylene Blue over nZVI/n-Lignin in Aqueous Solutions

For the removal ratio of methylene blue, 0.5 mL methylene blue solution was added to DI-H_2_O to give a total volume of 50 mL, with a concentration of 3 ppm. Forty milligrams each of the bare nZVI, EG-functionalized nZVI, and nZVI/n-lignin powders were added to the solution to start the degradation and adsorption of methylene blue dye. The absorption of the filtered solution after a certain duration was recorded by an UV–Vis spectrometer (Jasco, V-650, Hachioji, Tokyo, Japan) to monitor the degradation and adsorption process of methylene blue. The absorption intensity of the methylene blue solution at the wavelength of 664 nm decreased as the reaction time increased.

## 3. Results and Discussion

### 3.1. Characterization of Bare nZVI, EG-Functionalized nZVI, and nZVI/n-Lignin

**Figure 2:**
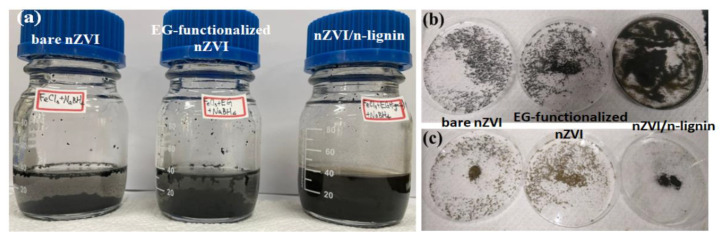
(**a**) After the synthesis for 30 min, the solution of the bare nZVI (left), EG-functionalized nZVI (middle), and nZVI/n-lignin (right). (**b**) After drying in the oven, the powders of the bare nZVI (left), EG-functionalized nZVI (middle), and nZVI/n-lignin (right). (**c**) After several weeks under atmospheric conditions, the powders of the bare nZVI (left), EG-functionalized nZVI (middle), and nZVI/n-lignin (right).

EG was selected to dissolve Kraft lignin, which could be subsequently converted to lignin nanoparticles simply by using the antisolvent precipitation or acid precipitation method. After the addition of the lignin dissolved in EG to the acidic H_2_O containing FeCl_3_ (pH ~ 2), nanoscale lignin was formed, suspended in the H_2_O. Once NaBH_4_ was added, tiny black particles (nZVI/n-lignin) were formed instantly. After the synthesis for 30 min, the solution with nZVI/n-lignin was still turbid, as shown in Figure 2a (right). For comparison, bare nZVI synthesized in H_2_O (Figure 2a, left) and EG-functionalized nZVI synthesized in H_2_O containing a small amount of EG (Figure 2a, middle) were also prepared. Iron particles in both the solution with bare nZVI and the solution with EG-functionalized nZVI aggregated and formed bigger clusters in H_2_O. To collect the powders of the nZVIs and nZVI/n-lignin, the three solutions were then washed three times with ethanol and put into an oven at a temperature of 40 °C for 20 min to evaporate the ethanol. The nZVI and nZVI/n-lignin powders are shown in Figure 2b. The bare nZVI (left) and EG-functionalized nZVI (middle) both show an obviously fractal pattern and are granular, but nZVI/n-lignin produced a very fine powder. Afterward, these three samples were placed for several weeks under atmospheric conditions. As Figure 2c shows, the powders of the bare nZVI (left) and EG-functionalized nZVI (middle) became yellowish, indicating their oxidation to form iron oxide. On the other hand, nZVI/n-lignin (right) maintained its black color and magnetic property, without obvious aggregation. These observations initially indicate that nanoscale lignin can prevent nZVI aggregation and protect nZVI from serious oxidation in the air.

**Figure 3:**
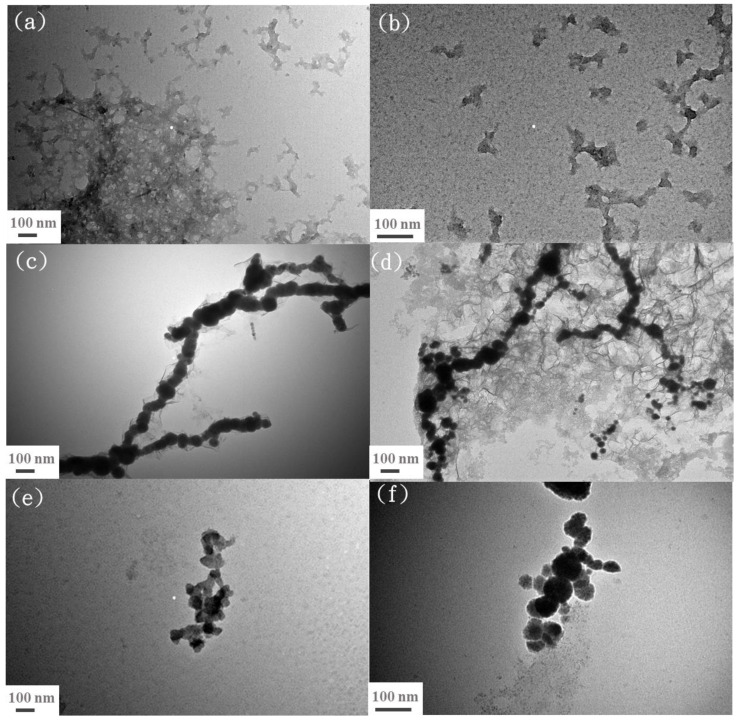
TEM images: (**a**) nanoscale lignin at a magnification of 100,000×, (**b**) nanoscale lignin at a magnification of 200,000× (**c**) bare nZVI at a magnification of 100,000×, (**d**) EG-functionalized nZVI at a magnification of 100,000×, (**e**) nZVI/n-lignin at a magnification of 100,000×, and (**f**) nZVI/n-lignin at a magnification of 200,000×.

For comparison with the nZVI/n-lignin composite, nanoscale lignin was first synthesized by the same procedure as was used for nZVI/n-lignin, described in Section 2.2, without the reduction step using NaBH_4_. TEM images displaying lignin clusters with a fractal shape are shown in Figure 3a. In Figure 3b, lignin with an irregular shape and a size of 100 nm is shown at higher magnification, which can prove the presence of nanoscale lignin in the mixed solutions of EG and H_2_O. Figure 3c,d are the TEM images of bare nZVI and EG-functionalized nZVI, respectively. Both nZVI materials are arranged in a chain morphology pattern and form large clusters, mainly due to the magnetic interactions of individual particles. For the TEM observation of the nZVI/n-lignin composite, the small ion nanoparticles gather into fractal nanoscale lignin as shown in Figure 3e. In the TEM image at a higher magnification, Figure 3f, the diameter of the nanoparticles is mostly less than 100 nm. The nZVI/n-lignin composite, in which nZVI particles are confined in the structure of nanoscale lignin, is a magnetic substance and has a smaller size than that of the bare and EG-functionalized nZVIs. In addition, the long-chain patterns of the bare and EG-functionalized nZVIs were not observed in the case of the nZVI/n-lignin composite, which can also explain its dispersibility in H_2_O.

**Figure 4:**
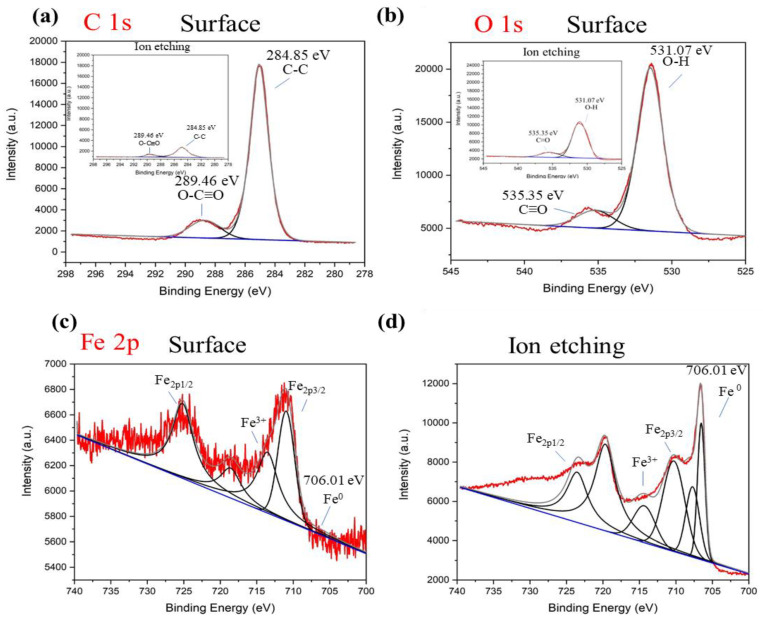
XPS analysis of nZVI/n-lignin: (**a**) C 1s scan, (**b**) O 1s scan, (**c**) Fe 2p scan, and (**d**) Fe 2p scan after argon ion etching. The insets in (**a**,**b**) are C 1s and O 1s of nZVI/n-lignin after argon ion etching.

To investigate the composition and chemical states of nZVI/n-lignin, XPS measurements of nZVI/n-lignin are shown in Figure 4. The XPS data were analyzed by the software Thermo Avantage. Two strong peaks were present for C 1s and O 1s, and a minor peak was observed for Fe 2p. Raw Kraft lignin showed strong peaks of binding energy around 285 eV for C 1s and 531 eV for O 1s due to the lignin structure containing the C–C, C–H, C-OH, C-O-C, O–O, Ph-OH, and Ph-C structures [24]. Obviously, the XPS spectra of nZVI/n-lignin for C 1s peaking at 285.53 eV (Figure 4a) and O 1s peaking at 531.03 eV (Figure 4b) are a result of the lignin structure, which contains three basic monolignol structures, coniferyl alcohol (G), sinapyl alcohol (S), and *p*-coumaryl alcohol (H). The photoelectron peaks for Fe 2p of nZVI/n-lignin are less intensive than those for C 1s and O 1s, as shown in Figure 4c. For the XPS spectrum of the bare nZVI, the peaks of binding energy at 710.56, 719.26, and 723.91 eV are for iron oxide and iron hydroxide [28], while the peak at 706.01 eV is for zero-valent iron [29]. As Figure 4c shows, only peaks for iron oxide and hydroxide were observed on the surface of nZVI/n-lignin, and there was no significant signal for zero-valent iron. To confirm the existence of zero-valence iron, argon ion etching on nZVI/n-lignin was performed during XPS analysis. A strong peak at 706.01 eV for zero-valent iron appeared, and the signal intensity for iron oxide and iron hydroxide at 710.56, 719.26, and 723.91 eV also increased in a similar manner to the XPS spectrum of Fe 2p, as shown in Figure 4d. Furthermore, the peak intensity for C 1s and O 1s decreased dramatically after ion etching, as shown in the insets of Figure 4a,b. Therefore, the XPS result indicates that the surface of nZVI is a thin layer of iron oxide/hydroxide and that the whole nZVI particle is surrounded by a lignin structure for providing steric stabilization. Desalegn et al. synthesized nZVI using mango peel extract [30]. XPS depth-profiling analysis by ion etching also shows increased peak intensity for nZVI around 706 eV.

**Figure 5:**
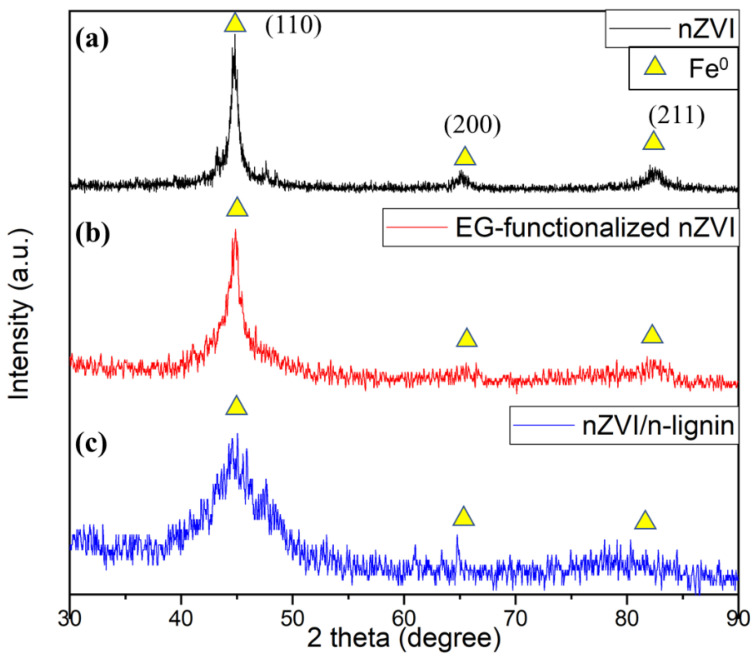
XRD analysis: (**a**) bare nZVI, (**b**) EG-functionalized nZVI, and (**c**) nZVI/n-lignin.

To identify the presence of nZVI in the lignin, XRD measurements of the bare nZVI, EG-functionalized nZVI, and nZVI/n-lignin were also conducted. A strong peak of lignin was observed at the 2θ scan of 29.4° (not shown). As Figure 5a shows, three peaks at the 2θ scan of 44.9°, 65.0° and 82.3° are clear for the XRD result of the bare nZVI, which indicate the iron crystal orientations of (110), (200), and (211) planes, respectively [31,32]. In Figure 5b, the noise of the XRD spectra increases due to the smaller size of the EG-functionalized nZVI. Moreover, the XRD analysis of nZVI/n-lignin is shown in Figure 5c. A broad peak in the noisy XRD spectrum is observed near 44.9°, indicating the presence of nano-sized α-Fe^0^ due to the formation of the composite with three-dimensional-branched lignin in the EG solution.

### 3.2. Dispersibility and Stability against Oxidation of nZVI/n-Lignin

**Figure 6:**
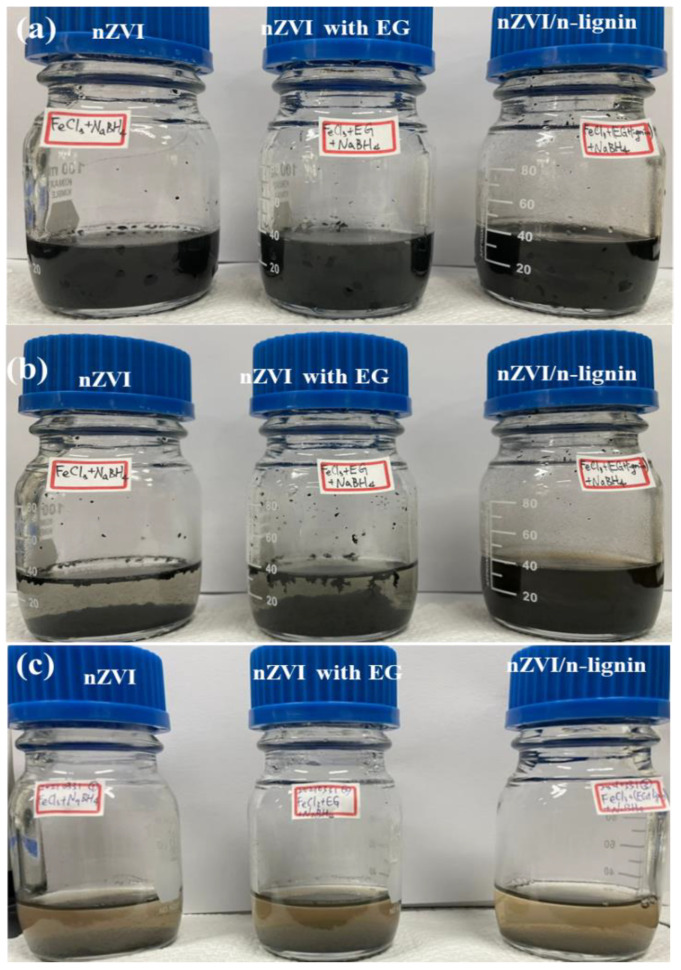
Dispersibility in H_2_O of bare nZVI, EG-functionalized nZVI, and nZVI/n-lignin: (**a**) after the sonication, (**b**) standing for 60 min, and (**c**) standing for 1 day.

The dispersibility of the bare nZVI, EG-functionalized nZVI and nZVI/n-lignin in aqueous solutions over different durations was tested. After the synthesis and washing processes, the three samples were put into water and dispersed by an ultrasonicator. After sonication for a short time, optical images of the solutions were taken, as shown in Figure 6a. The results for the solutions after standing for 60 min are shown in Figure 6b. The bare and EG-functionalized nZVIs formed large clusters that reduced their dispersibility in H_2_O. However, the solution with nZVI/n-lignin maintained its black color, which indicates that nZVI/n-lignin was still well dispersed and suspended in the H_2_O. The results for the solutions after standing for one day are shown in Figure 6c. The solutions became yellowish, indicating the oxidation of nZVIs. When nZVIs are put into water, they start to be oxidized by H_2_O/O_2_ to form iron oxide/hydroxide according to the following equations [31,32]: (1) Fe^0^ + 2H_2_O → Fe^2+^ + H_2_ + 2OH^−^; (2) 2Fe^0^ + O_2_ + 2H_2_O → 2Fe^2+^ + 4OH^−^; (3) 6Fe^2+^ + O_2_ + 6H_2_O → 2Fe_3_O_4(s)_ + 12H^+^; (4) Fe^2+^ + 2OH^−^ → Fe(OH)_2(s)_; (5) 6Fe(OH)_2(s)_ + O_2_ → 2Fe_3_O_4_ + 6H_2_O. According to our observations, obvious precipitation of the solution with nZVI/n-lignin only happened after it had been standing for several days. The result shows that nZVI/n-lignin possesses better dispersibility in H_2_O than that of the bare nZVI and EG-functionalized nZVI, probably because ion nanoparticles imbed in nano-sized lignin to prevent the formation of a chain-like pattern.

**Figure 7:**
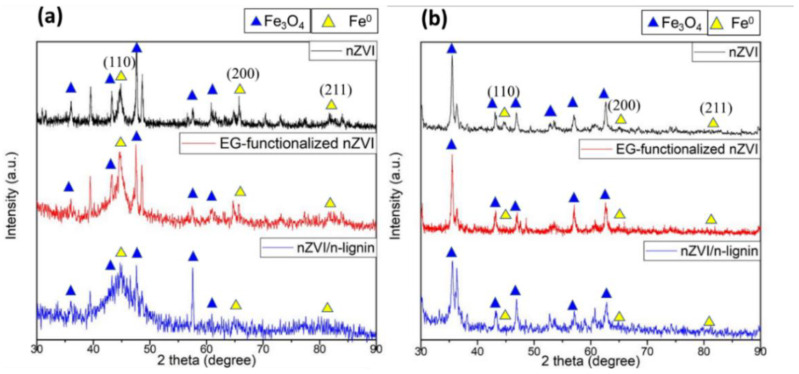
XRD analysis of aged bare nZVI, EG-functionalized nZVI, and nZVI/n-lignin: (**a**) exposed to air for 7 days and (**b**) put in water for 7 days.

After the synthesis, the three dried materials were stored in atmospheric conditions for 7 days. Then, the powders were analyzed by XRD. The XRD measurements of the three aged materials in atmospheric conditions are shown in Figure 7a. The peak intensity of the nZVIs decreased dramatically, especially for the bare and EG-functionalized nZVIs, because of the oxidation of nZVI in the air. The XRD of the nZVI/n-lignin stored in atmospheric conditions for 7 days did not change, which indicates the better stability against oxidation of nZVI/n-lignin in the air. In a separate experiment, the three materials after synthesis were placed in H_2_O for 7 days. The XRD analysis of the three aged materials placed in H_2_O for 7 days is shown in Figure 7b. All the peaks from the nZVIs disappeared and were replaced by peaks at 35.52°, 43.17°, 47.27°, 53.56°, 57.10°, and 62.7°, indicating the formation of Fe_3_O_4_. These characteristic peaks in the X-ray diffraction indicate the crystal orientations of (311), (400), (331), (422), (511), and (440), respectively. The XRD results show the better stability against oxidation of nZVI/n-lignin and the oxidation reaction of nZVIs to iron oxide.

### 3.3. Removal of Methylene Blue in Aqueous Solutions

**Figure 8:**
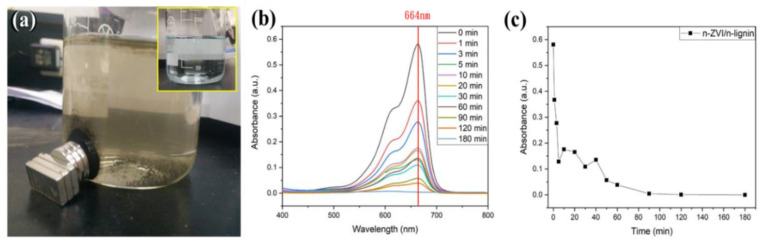
(**a**) The image of the degradation of methylene blue in aqueous solutions by nZVI/n-lignin. The inset is the image before the addition of nZVI/n-lignin. (**b**) UV–Vis spectra of high ratio test and (**c**) the absorption as a function of time at the wavelength of 664 nm.

Besides the synthesis, the dispersibility in water, and the antioxidation in the air of nZVI/n-lignin, the removal of methylene blue in H_2_O by nZVI/n-lignin was also investigated. In the presence of nZVIs, the removal mechanisms of methylene blue (MB) include reduction into colorless leuco-MB, precipitation as Fe(II)-MB, absorption as ZVI-MB, and degradation using hydroxyl radicals [33]. Moreover, the methylene blue dye in the solution can be adsorbed by biopolymers such as lignin and chitosan [10,34]. The absorption mechanism for dyes and lignin may involve electrostatic interactions between the MB molecule with positively charged nitrogen and the dissociated functional groups of lignin [35]. Therefore, the inset of Figure 8a shows an image of the methylene blue aqueous solutions. After 40 mg of nZVI/n-lignin was added to the methylene blue solution and stirred vigorously with a magnetic bar, the color of the solution quickly faded within a short time until a yellow color appeared, indicating the removal of the methylene blue as shown in Figure 8a. The decolorization of methylene blue from blue to yellow is consistent with reported studies [36]. The removal ratio of methylene blue can be monitored by a UV–Vis spectrometer. The UV–Vis spectrum of the methylene blue in H_2_O had a strong absorption peak at the wavelength of 664 nm and a shoulder peak at around 615 nm from the dimer of methylene blue [33]. The UV–Vis spectra between 400 and 800 nm of the methylene blue degraded and adsorbed by the nZVI/n-lignin composite in H_2_O are shown in Figure 8b. The UV–Vis spectra of methylene blue gradually decreased to no absorption, indicating that it had been fully removed by nZVI/n-lignin. The absorption of the solutions at 664 nm as a function of time is shown in Figure 8c. Two regions are observed. In the first region (0~10 min), the removal rate of methylene blue, including degradation and adsorption, is very fast. After 30 min, the removal rate becomes slower, and methylene blue is removed completely after 90 min. The reason for the slower decreasing rate and fluctuation in the second region may come from the degradation product and iron oxidation product diffusing throughout the lignin structure, interfering in the contact between the methylene blue and nZVI within the lignin structure.

**Figure 9:**
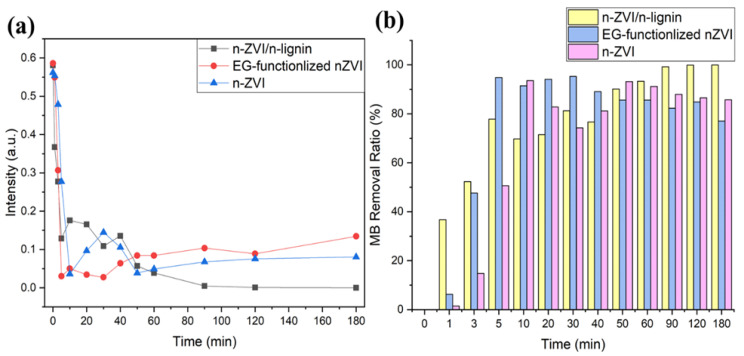
(**a**) UV–Vis absorption intensity at the wavelength of 664 nm for bare nZVI, EG-functionalized nZVI, and nZVI/n-lignin. (**b**) The removal ratio of methylene blue for bare nZVI, EG-functionalized nZVI, and nZVI/n-lignin.

In practical application, once the nZVI is added to ground water in the field for pollutant remediation, the oxidation reaction between nZVI and H_2_O/O_2_ starts, which consumes the nZVI before it degrades the pollutants. The nZVI’s stability against H_2_O/O_2_ oxidation is of great importance. In our study, the applicability of bare nZVI, EG-functionalized nZVI, and nZVI/n-lignin was compared according to the removal ratio of methylene blue in aqueous solutions. After the addition of 40 mg each of bare nZVI, EG-functionalized nZVI, and nZVI/n-lignin to the methylene blue solution, their absorption rates were measured by UV–Vis spectra, as shown in Figure 9a. The two nZVIs and nZVI/n-lignin could remove methylene as the duration increased. Of the three, the reaction of the 40 mg nZVI/n-lignin composite could persevere for the longest duration, although just a small amount of nZVI is in nanoscale lignin. On the other hand, from the removal ratio of methylene blue shown in Figure 9b, nZVI/n-lignin performed best in the removal of methylene blue in aqueous solutions. This is because of the better dispersibility in water and stability against oxidation of the nZVI/n-lignin composite.

## 4. Conclusions

In our study, sustainable lignin biopolymer was employed for the synthesis of iron nanoparticles for the removal of environmental pollutants. nZVI is the most frequently studied material for the water treatment aspect of environmental engineering. In real field applications, the environmental pollutants in a contaminated area are far away from the nZVI injection sites. Consequently, the prevention of nZVI aggregation and the avoidance of strong oxidation by H_2_O/O_2_ are of great importance. The agglomeration of the nZVI results in reduced degradation activity and poor mobility. Strongly oxidized behavior results in the consumption of the nZVI before it makes contact with the pollutants away from the injection sites. In this study, the nanoscale lignin-protected nZVI (nZVI/n-lignin) was first proposed to solve the above-mentioned issues. According to the analysis of the TEM and XPS measurements, the structure of nZVI/n-lignin is nanoscale lignin surrounding nZVI particles, which consist of zero-valent iron as the core with a thin layer of defective iron oxide/hydroxide on the surface. The traditional chain-like pattern and large clusters of bare nZVI can be avoided in the case of nZVI/n-lignin probably because of the steric stabilization alleviating magnetic interactions. This feature, combined with the use of nano-sized lignin rather than the use of micro-sized lignin, makes nZVI/n-lignin possess much better dispersibility and mobility in H_2_O than those of bare nZVI. The structure of nano-lignin could provide more heterogeneous nucleation sites for the growth of ion nanoparticles. From their optical images, TEM, and XRD analysis, it was observed that nZVI/n-lignin possesses better stability against oxidation by H_2_O/O_2_ than those of bare and EG-functionalized nZVIs. The as-synthesized nZVI/n-lignin maintains its degradation ability even after it has been stored for several weeks in atmospheric conditions or several hours in H_2_O, while bare nZVI is quickly oxidized to form iron oxide/hydroxide in a much shorter time. Compared with nZVIs, the nZVI/n-lignin composite may have better mobility in ground water while maintaining its degradation ability toward pollutants far away from the injection sites. In summary, the presented synthetic method for nZVI/n-lignin involving a dissolution and precipitation approach with ethylene glycol/acidic H_2_O is easy to carry out, and it is environmentally friendly. It could be used as a universal approach for synthesizing nanoscale lignin-stabilized zero-valent metal and metal oxide nanoparticles for various applications.

## Data Availability

The data presented in this study are available on request from the corresponding author.
